# Obesity with radiological changes or depression was associated with worse knee outcome in general population: a cluster analysis in the Nagahama study

**DOI:** 10.1186/s13075-020-02375-w

**Published:** 2020-11-27

**Authors:** Kazuya Nigoro, Hiromu Ito, Tomotoshi Kawata, Kohei Nishitani, Yasuharu Tabara, Fumihiko Matsuda, Shu Narumiya, Shuichi Matsuda, on behalf of the Nagahama Study group

**Affiliations:** 1 Department of Drug Discovery Medicine, Kyoto University Graduate School of Medicine, Kyoto, Japan; 2 Department of Orthopaedic Surgery, Kyoto University Graduate School of Medicine, Kyoto, Japan; 3 Department of Advanced Medicine for Rheumatic Diseases, Kyoto University Graduate School of Medicine, Kyoto, Japan; 4 Center for Genomic Medicine, Kyoto University Graduate School of Medicine, Kyoto, Japan

**Keywords:** Osteoarthritis, Knee pain, Cluster analysis, Phenotype

## Abstract

**Background:**

In knee osteoarthritis (OA), pain is the most frequent and dominant symptom. However, which factors other than radiological changes contribute to the symptoms is unresolved. The aims of this study were to identify factors affecting knee pain from various variables with radiological changes taken into count and exploratively examine what subgroups or phenotype could be identified by cluster analysis using the identified knee pain factors.

**Methods:**

Patients 60 years or older who underwent radiographic evaluation were included in this cross-sectional study, and those subjects who completed a questionnaire about knee symptoms without missing data were eligible for analysis. Multiple regression analysis was used to examine the associations between selected variables and The Japanese Knee Osteoarthritis Measure (JKOM) pain score. We grouped the subjects by cluster analysis using identified variables.

**Results:**

Two thousand five hundred forty-two subjects were included in the full set of analyses. Age, body mass index (BMI), radiological grade, bone mineral density (BMD), and high-sensitivity C-reactive protein (hs-CRP) showed a statistically significant correlation with radiological showing the strongest value. For dichotomous variable, presence of depression showed a statistically significant result. We used BMI, radiological grade, BMD, hs-CRP, and presence of depression as a variable for cluster analysis and identified six subgroups: (1) minimal joint disease subgroup, (2) male and high BMD subgroup, (3) high CRP subgroup, (4) severe radiological OA subgroup, (5) depressive subgroup, and (6) moderate radiological OA with high BMI subgroup, showing the worst knee outcome.

**Conclusion:**

This study identified the factors affecting knee pain other than radiological changes and identified six subgroups of knee outcome in the general population. The results showed that obesity with radiological changes or depression was associated with worse knee outcome.

## Background

Knee osteoarthritis (OA) is one of the most prevalent and troublesome musculoskeletal diseases in the older population. In knee OA, pain is the most frequent and dominant symptom and the major reason for clinical decision-making [1]. It is known, however, that the radiologically defined structural severity of knee OA has a relatively low specificity for explaining knee pain or symptoms [1–3], and which factors other than radiological changes contribute to the symptoms is unresolved. Such analyses are required to allow the application of appropriate treatment or preventative measures to each patient or subject in clinical practice and in administrative decision-making.

Knee OA and its symptoms involve a wide array of contributing factors that affect the appearance and course of the disease, such as genetic background, obesity, muscle weakness, mechanical misalignment, degenerative changes of the articular cartilage, inflammation in the tissues including synovia, and subchondral bone changes [1–4]. In addition, knee pain has been shown to be influenced by factors that are not specific to the knee joint, such as the patient’s psychopathological features and changes in neural sensitization [5–7]. For these reasons, it has become clearer that knee OA is a heterogeneous disease, and some consider to be a syndrome rather than a disease [1, 8].

Although several studies have been published on the factors associated with knee pain, there are limited reports that have comprehensively examined the factors thought to be associated with knee pain in one study. Therefore, the aims of this study were to identify factors affecting knee pain and to find weighted contributions from various variables. To address these aims, this study took advantage of a large community-based cohort that included almost 10,000 individuals for whom a range of demographic and specific data including biomarkers was available. Additionally, attempts have been made to classify knee OA into several subgroups or phenotypes based on clinical appearance and associated factors [5, 9]. As few studies conducted analysis from a multidimensional point of view in a large number of subjects [5], we exploratively examined what subgroups or phenotype could be identified by cluster analysis using the identified knee pain factors.

## Methods

### Study population

This cross-sectional study used data from the Nagahama Prospective Cohort for Comprehensive Human Bioscience (the Nagahama Study). The Nagahama study consists of 9850 middle-aged to elderly citizens who were recruited from 2013 to 2016 from the general population living in Nagahama City, a largely rural city of 125,000 inhabitants located in central Japan. Residents aged 34 to 80 years in the community who were able to participate independently and no serious disease or symptom or health problem were recruited. The details of recruitment of participants have been reported elsewhere [10].

Participants 60 years or older who underwent radiographic evaluation were included, and those subjects who completed a questionnaire about knee-related outcomes without missing data were eligible for the analysis.

This study was conducted according to the principles of the Declaration of Helsinki and was approved by the ethics committee of Kyoto University Graduate School of Medicine and by the Nagahama Municipal Review Board (No. C278). Written informed consent was obtained from all participants.

### Variables examined

We collected data from participants who agreed to participate in this particular study. The radiological severity of knee OA was evaluated as Kellgren/Lawrence (K/L) grade [11]. Both knees were evaluated by two registered orthopedic surgeons, and the higher score for the knee joints was used for analysis. The knees on which any replacement surgery performed were identified in the X-ray and excluded. For evaluation of skeletal muscle mass, a multi-frequency electrical impedance meter (InBody 430, Biospace Japan, Tokyo, Japan) was used. Knee extension strength was measured by sitting position on a chair with 90° flexion of the hip and knee joints using a dynamometer (Musculater, OG Giken Co., Okayama, Japan). For assessment of bone mineral density (BMD), subjects underwent calcaneal quantitative ultrasound (Benus α ultrasound device; Nihon Kohden; Tokyo, Japan) and the measured *T* score was used for analysis [12]. Depressive symptoms were assessed based on the score for the Mental Health Inventory-5 (MHI-5) [13]. The validity of using MHI-5 in Japanese people has previously been reported, and subjects with a score ≤ 52 are considered to have depressive symptoms [14]. For inflammatory biomarker, high-sensitivity C-reactive protein (hs-CRP) were measured (reagent: CardioPhase hs-CRP; instrumentation: BN II system; Siemens). We converted any undetectable values of hs-CRP (≤ 0.05 μg/ml, 23 subjects) to 0.05 μg/ml for the analysis. The value of hs-CRP was logarithmically transformed when statistical analysis was performed. Hemoglobin A1c (HbA1c) was measured by the latex agglutination method (Detaminar L-HbA1c; Kyowa Medex Co., Ltd., Tokyo, Japan) and was estimated as a National Glycohemoglobin Standardization Program (NGSP) equivalent value (%). High-density lipoprotein-cholesterol (HDL-C) and triglyceride levels were measured by the enzymatic assay (Metaboread-LDL or Detaminar C-TG; Kyowa Medex Co., Ltd., Tokyo, Japan). Low-density lipoprotein-cholesterol (LDL-C) levels and uric acid levels were measured by the homogeneous method (Detaminar L-HDL-C; Kyowa Medex Co., Ltd., Tokyo, Japan) and the uricase-POD method (Detaminar C-UA; Kyowa Medex Co., Ltd., Tokyo, Japan), respectively.

Any medical history of the knee surgery was reported, and the information on the use of analgesics and/or oral steroids was collected irrespective of the aim of the use.

### Clinical outcome

We used the Japanese Knee Osteoarthritis Measure (JKOM), which is a validated outcome measure for Japanese patients with knee OA [15], to identify knee-related outcomes in the participants. JKOM consists of four main items: pain and stiffness (pain; a total of eight questions, 0–32 points), activities of daily living (ADL; a total of 10 questions, 0–40 points), participation in social activities (social activity; a total of five questions, 0–20 points), and general health conditions (general health; a total of two questions, 0–8 points) with 100 points in total score as the maximum. Higher JKOM score indicates worse condition. In a previous study, JKOM showed reliability and validity for clinical outcomes in comparison with other health-related scales, such as the Western Ontario and McMaster Universities Arthritis Index (WOMAC) and the Medical Outcomes Study 36-Item Short-Form Health Survey (SF-36) [15]. All participants self-reported their responses to the JKOM questionnaire.

### Statistical analyses

Descriptive statistics are reported as mean and standard deviation (SD) for continuous variables and as a proportion for dichotomous variables.

To examine the associations between each variable and JKOM pain score, Pearson’s correlations were used for continuous variables and Student’s *t* test were used for dichotomous variables. Multiple regression analysis was used to examine the associations between selected variables and JKOM pain score. The starting model (full model analysis) included all variables for the analysis. We selected the variable which showed a statistically significant correlation with JKOM pain for reduced model 1. For reduced model 2, variables were selected by stepwise method. For reduced model 3, we excluded age from reduced model 2.

Cluster analysis is one of the most commonly used analytical method in OA field [5], and K-means cluster analysis [16] was performed to identify the phenotypes for this study using variables which were identified from the multiple regression analysis. We attempted clustering into three to seven groups. The best-fit number of clusters was determined by the highest cubic clustering criterion (CCC) value. The cluster analysis was validated by means of repeated cluster analyses using randomly selected data from the whole data set [17].

Differences in JKOM scores between the subgroups were compared using analysis of covariance. The JKOM scores of pairs of subgroups were compared using Student’s *t* test. Adjustment was made with age, sex, duration of knee symptoms, and knee extension strength in order to validate the score differences among the subgroup clusters. The threshold for significance was *P* < 0.05. All statistical analyses were conducted using JMP Pro (v. 14.0.0; SAS Institute, Cary, NC, USA). Cluster analyses were inspected and validated by Satista Co. Ltd. (https://www.satista.jp/medical/).

## Results

### Study population

**Fig. 1:**
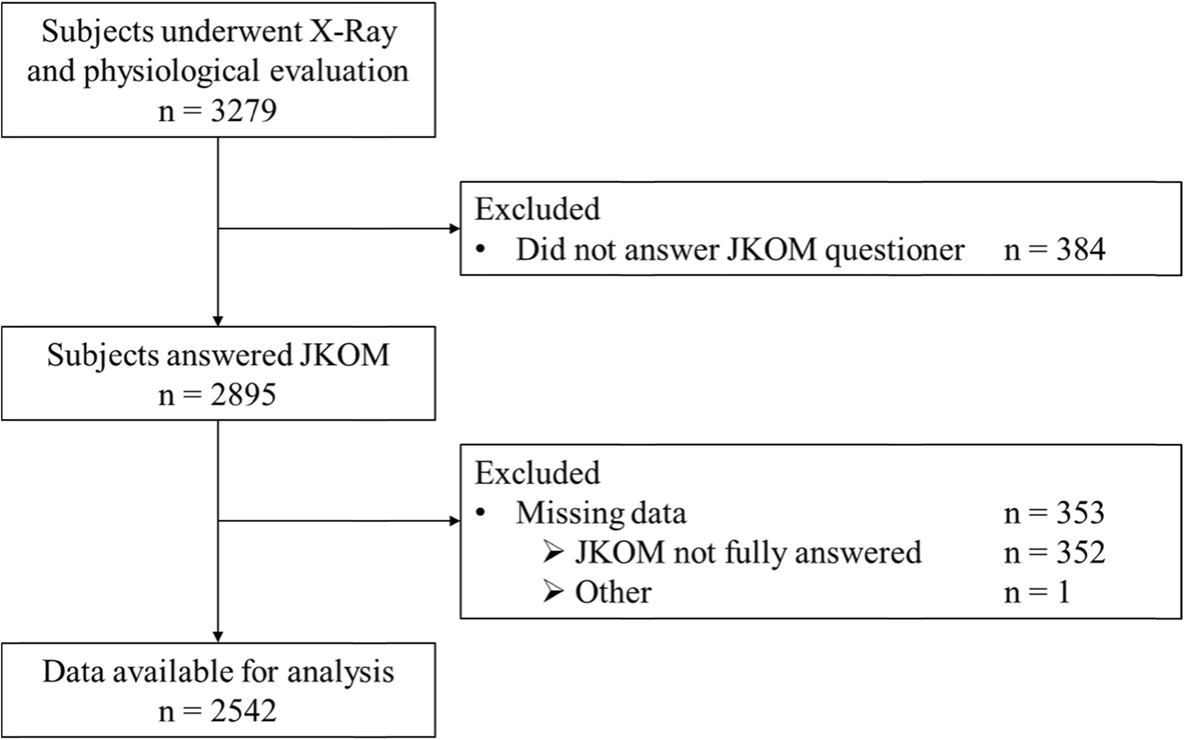
Flow diagram of study participants. JKOM, Japanese Knee Osteoarthritis Measure

A total of 9850 participants were assessed for eligibility in this study. Participants aged 60 or older (*n* = 5018) were included in the first surveillance of this study and were asked to undergo further radiographic (knee X-ray) and physiological (muscle strength) evaluation; 1739 subjects declined. The remaining 3279 subjects agreed to participate in this particular study. After the full set of data was surveyed, 384 participants who did not complete the JKOM questionnaire were excluded from further analyses. Of the remaining subjects, 353 with missing data were excluded. The remaining 2542 subjects were included in the full set of analyses (Fig. 1). Only five participants reported the previous knee surgery other than replacement arthroplasty and were included in this analysis. Data for knee extension strength were available for 1337 subjects and used in a sub-analysis. Table 1 shows the characteristics of all study subjects.

**Table 1:** Characteristics of study participants

Variable	Analysis population (*n* = 2542)
Age, years	68.7 (5.2)
Female, *n*, %	1625, 63.9
BMI, kg/m^2^	22.4 (3.1)
Radiographic severity, *n*, %	
K/L grade 0	103, 4.1
K/L grade 1	1465, 57.6
K/L grade 2	735, 28.9
K/L grade 3	216, 8.5
K/L grade 4	23, 0.9

Values are mean (SD) except where otherwise indicated

*BMI* body mass index, *K/L* Kellgren/Lawrence

### Relationship between each variable and JKOM pain

Table 2 shows the correlation between each variable and JKOM knee pain. Age, BMI, K/L grade, BMD, hs-CRP, Hb-A1c, and HDL-Cho showed a statistically significant correlation with K/L grade showing the strongest value (correlation coefficient; 0.391). For dichotomous variable, depression showed statistically significant results, but sex did not. Next, we conducted multiple regression analysis (Table 3). Age, BMI, K/L grade, and depressive symptoms were unanimously showed as independent variables in full mode and reduced models 1 and 2. For clinical practice and epidemiologic studies, phenotypic distinctions should be confined to those that affect decisions about treatment or prevention [9, 18, 19]. Although age is a known factor related with knee OA, we conducted the analysis without age for reduced model 3. In this model, BMD and hs-CRP statistically showed independent effects on JKOM pain in addition to BMI, K/L grade, and depression. We decided to use these 5 variables for the cluster analysis.

**Table 2:** Correlation between each variable and JKOM pain score

	Correlation coefficient	95% CI	*P* value
**Age**	**0.176**	**0.138–0.214**	**< 0.0001**
Sex*	**–**	**–**	0.0571
**BMI**	**0.206**	**0.169–0.243**	**< 0.0001**
**K/L grade**	**0.391**	**0.358–0.424**	**< 0.0001**
Muscle mass of lower extremity	− 0.011	− 0.050–0.028	0.5706
**BMD**	**− 0.047**	**− 0.086 to − 0.008**	**0.0173**
Depressive symptom*	**–**	**–**	**0.0100**
**hs-CRP**	**0.093**	**0.055–0.132**	**< 0.0001**
**Hb-A1c**	**0.056**	**0.017–0.095**	**0.0049**
**HDL-cholesterol**	**− 0.060**	**− 0.098 to − 0.021**	**0.0027**
LDL-cholesterol	− 0.031	− 0.070–0.008	0.1197
Triglyceride	0.014	− 0.025–0.053	0.4827
Uric acid	0.021	− 0.018–0.059	0.3016

Pearson’s correlations were used for analysis

Variables in bold indicate statistically significant results

*BMI* body mass index, *BMD* bone mineral density, *hs-CRP* high-sensitivity C-reactive protein, *HbA1c* hemoglobin A1c, *K/L* Kellgren/Lawrence

*Student’s *t* test was used for dichotomous variables

**Table 3:** Results of multiple regression analyses of each variable and JKOM pain score in each model

	Full model		Reduced model 1		Reduced model 2		Reduced model 3	
	Standardized beta (95% CI)	*P* value	Standardized beta (95% CI)	*P* value	Standardized beta (95% CI)	*P* value	Standardized beta (95% CI)	*P* value
Age	**0.096 (0.052–0.129)**	**< 0.0001**	**0.106 (0.065–0.134)**	**< 0.0001**	**0.105 (0.065–0.133)**	**< 0.0001**	**–**	**–**
Sex	− 0.038 (− 0.0543– 0.156)	0.2772	**–**	**–**	–	**–**	**–**	**–**
BMI	**0.136 (0.146–0.284)**	**< 0.0001**	**0.128 (0.139–0.26)**	**< 0.0001**	**0.124 (0.135–0.256)**	**< 0.0001**	**0.123 (0.133–0.255)**	**< 0.0001**
K/L grade	**0.344 (1.999–2.509)**	**< 0.0001**	**0.340 (1.977–2.460)**	**< 0.0001**	**0.340 (1.982–2.465)**	**< 0.0001**	**0.359 (2.104–2.583)**	**< 0.0001**
Muscle mass of lower extremity	− 0.020 (− 0.309–0.168)	0.5634	–	–	–	–	–	–
BMD	− 0.030 (− 0.346–0.390)	0.1187	− 0.025 (− 0.312–0.057)	0.1763	− 0.026 (− 0.314–0.054)	0.1660	**− 0.038 (− 0.376 to − 0.008)**	**0.0412**
Depressive symptom	**0.068 (0.274–0.875)**	**0.0002**	**0.067 (0.268–0.867)**	**0.0002**	**0.067 (0.272–0.870)**	**0.0002**	**0.064 (0.241–0.843)**	**0.0004**
hs-CRP	0.033 (− 0.055–0.749)	0.091	0.033 (− 0.050–0.743)	0.0864	0.030 (− 0.071–0.704)	0.1094	**0.037 (0.003–0.781)**	**0.0484**
Hb-A1c	− 0.001 (− 0.367–0.350)	0.9628	− 0.001 (− 0.349–0.365)	0.9649	**–**	–	**–**	**–**
HDL-cholesterol	0.010 (− 0.009–0.015)	0.6405	0.015 (− 0.007–0.015)	0.4626	**–**	–	**–**	**–**
LDL-cholesterol	− 0.018 (− 0.009–0.015)	0.3365	–	–	–	–	**–**	**–**
Triglyceride	− 0.014 (− 0.005–0.003)	0.5066	–	–	–	–	**–**	**–**
Uric acid	− 0.012 (− 0.212–0.119)	0.5814	–	–	–	–	**–**	**–**

Reduced model 1: Selected the variable which showed a statistically significant correlation with JKOM pain. Reduced model 2: Selected the variable by stepwise method. Reduced model 3: Excluded age from reduced model 2

Variables in bold indicate statistically significant results

*BMI* body mass index, *K/L* Kellgren/Lawrence, *BMD* bone mineral density, *hs-CRP* high-sensitivity C-reactive protein, *HbA1c* hemoglobin A1c

### Determination of the number of clusters

The choice of six clusters appeared to be best for this analysis because it gave the highest CCC score (Supplementary Table 1). The validity of the cluster analysis was also verified since we randomly split the entire data set into halves and carried out two independent cluster analyses and found the two sets of the clusters were very much similar. Both data set showed the highest CCC score for six clusters, and more than 95% of the subjects were classified into the same cluster as using the total data set (data not shown).

### Comparison of phenotypes

**Fig. 2:**
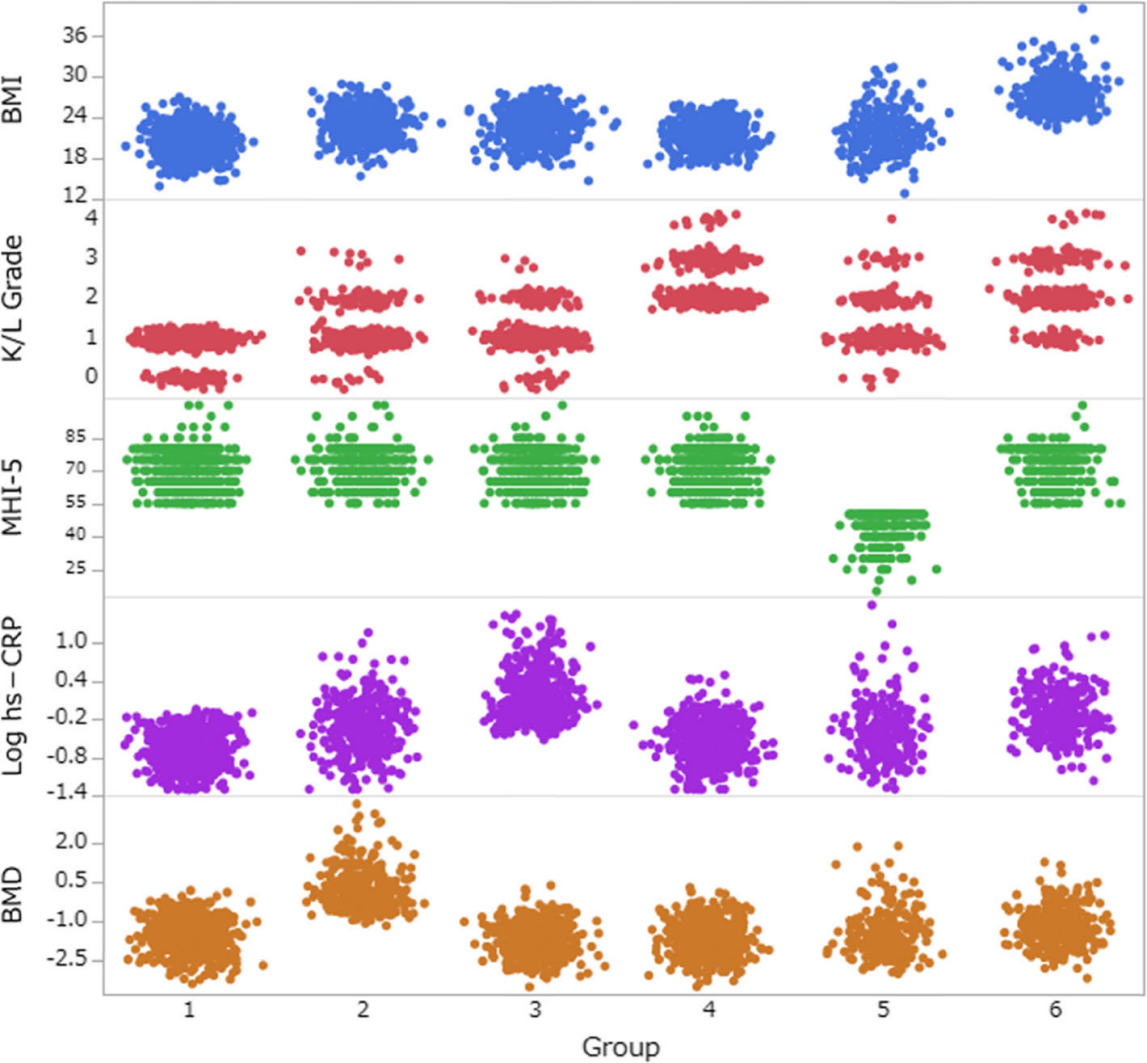
Scatter plots of each data in classified six subgroups. Distribution of variables used for cluster analysis in each group was visualized by scatter plots. JMP Pro 14.0.0 software was used. BMI, body mass index; K/L, Kellgren/Lawrence; MHI-5, Mental Health Inventory-5; hs-CRP, high-sensitivity C-reactive protein; BMD, bone mineral density

The characteristics of the six subgroups are summarized in Table 4 and are visualized by scatter plots in Fig. 2. The six subgroups were characterized and defined by (1) lower BMI, mild or no evidence of radiographic knee OA (ROA), and lower hs-CRP (minimal joint disease subgroup); (2) dominantly male, mild or moderate ROA, and higher T-score (male and high BMD subgroup); (3) mild or moderate ROA and higher hs-CRP (high CRP subgroup); (4) most severe ROA (K/L grade ≥ 2) and lowest T-score (severe ROA subgroup); (5) moderate ROA and severe depression (depressive subgroup); and (6) higher BMI and moderate or severe ROA (moderate ROA with high BMI subgroup).

**Table 4:** Characteristics of subgroups

Variable	Group 1 (*n* = 680)	Group 2 (*n* = 388)	Group 3 (*n* = 457)	Group 4 (*n* = 500)	Group 5 (*n* = 229)	Group 6 (*n* = 288)	Total (*n* = 2542)
Age, years	68.1 (5.1)	67.8 (5.0)	68.8 (5.2)	69.9 (5.2)	68.2 (5.2)	69.4 (5.08)	68.7 (5.2)
Female, *n*, %	439, 64.6	136, 35.1	253, 55.4	421, 84.2	176, 76.9	200, 69.4	1625, 63.9
BMI, kg/m^2^	20.5 (2.1)	22.9 (2.3)	22.7 (2.3)	21.5 (2.0)	21.7 (3.2)	27.4 (2.6)	22.4 (3.1)
Radiographic severity, *n*, %							
K/L grade 0	61, 9.0	13, 3.4	21, 4.6	0, 0	8, 3.5	0, 0	103, 4.1
K/L grade 1	619, 91.0	291, 75.0	369, 80.7	0, 0	140, 61.1	46, 16.0	1465, 57.6
K/L grade 2	0, 0	76, 19.6	63, 13.8	371, 74.2	62, 27.1	163, 56.6	735, 28.9
K/L grade 3	0, 0	8, 2.1	4, 0.9	114, 22.8	18, 7.9	72, 25.0	216, 8.5
K/L grade 4	0, 0	0, 0	0, 0	15, 3.0	1, 0.4	7, 2.4	23, 0.9
MHI-5 score	71 (8.1)	73 (7.9)	71 (8.4)	71 (8.3)	44 (7.5)	72 (8.6)	69 (11.3)
Depressive symptoms, *n*, %	0, 0	0, 0	0, 0	0, 0	229, 100	0, 0	229, 9.0
hs-CRP mean (SD), median, μg/ml	0.26 (0.16), 0.22	0.77 (1.17), 0.48	2.43 (3.71), 1.14	0.38 (0.35), 0.27	0.93 (3.05), 0.38	1.14 (1.52), 0.66	0.91 (2.1), 0.41
BMD (*T* score)	− 1.6 (0.63)	0.21 (0.76)	− 1.7 (0.62)	− 1.7 (0.68)	− 1.4 (0.89)	− 1.2 (0.72)	− 1.3 (0.95)

Values are mean (SD), except where otherwise indicated

Percentages may not sum to 100 because of rounding

Scores on the MHI-5 range from 0 to 100, with higher scores indicating better mental health. MHI-5 ≤ 52 were considered as having depressive symptoms

Defined groups are 1 = “minimal joint disease subgroup,” 2 = “male and high BMD subgroup,” 3 = “high CRP subgroup,” 4 = “severe ROA subgroup,” 5 = “depressive subgroup,” and 6 = “moderate ROA with high BMI subgroup”

*BMD* bone mineral density, *BMI* body mass index, *hs-CRP* high-sensitivity C-reactive protein, *K/L* Kellgren/Lawrence, *MHI-5* Mental Health Inventory-5, *ROA* radiographic knee OA

### Clinical outcomes

**Fig. 3:**
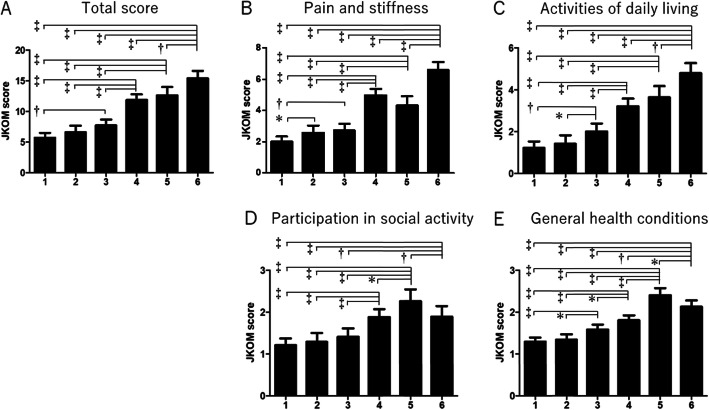
Comparison of JKOM among subgroups. Higher JKOM score indicates worse condition. **a** Total score. **b** Pain and stiffness. **c** Activities of daily living. **d** Participation in social activities. **e** General health conditions. Bars show the mean and 95% confidence interval. *P* values were calculated using Student’s *t* test. *p < 0.05, ^†^*p* < 0.005, ^‡^ *p* < 0.001. JKOM, Japanese Knee Osteoarthritis Measure

The comparative analysis of the total score for the six subgroups indicated that these subgroups could be divided into two major groups: milder knee symptom (groups 1–3) and more severe symptom subgroups (groups 4–6) (Fig. 3a, Table 5). Among the milder symptom subgroups, group 3 showed more symptoms than the other two subgroups (groups 1 and 2). Among the more severe symptom subgroups, group 6 showed the worst symptoms (compared with groups 4 and 5). The same trend was observed after adjustment for sex, age, duration of knee symptoms, and knee extension strength (Table 6).

**Table 5:** JKOM value of each subgroup

JKOM scores	Group 1 (*n* = 680)	Group 2 (*n* = 388)	Group 3 (*n* = 457)	Group 4 (*n* = 500)	Group 5 (*n* = 229)	Group 6 (*n* = 288)	Total (*n* = 2542)	*P* value
Pain and stiffness	2.0 (3.5)	2.6 (3.5)	2.7 (3.8)	5.0 (5.8)	4.3 (5.2)	6.6 (6.3)	3.5 (4.9)	< 0.0001
Activities of daily living	1.2 (2.7)	1.4 (2.9)	2.0 (3.7)	3.2 (5.1)	3.6 (5.3)	4.8 (6.0)	2.4 (4.3)	< 0.0001
Participation in social activities	1.2 (1.6)	1.3 (1.6)	1.4 (1.8)	1.9 (2.7)	2.3 (2.9)	1.9 (2.6)	1.6 (2.2)	< 0.0001
General health conditions	1.3 (1.2)	1.3 (1.2)	1.6 (1.3)	1.8 (1.4)	2.4 (1.6)	2.1 (1.5)	1.7 (1.4)	< 0.0001
Total score	5.7 (7.3)	6.6 (7.5)	7.7 (8.6)	11.9 (13.3)	12.6 (13.0)	15.4 (14.8)	9.2 (11.1)	< 0.0001

Values are mean (SD). Higher JKOM score indicates worse condition. *P* values were calculated using ANOVA

*JKOM* Japanese Knee Osteoarthritis Measure

**Table 6:** JKOM value of each subgroup after adjustment

JKOM scores	Group 1		Group 2		Group 3		Group 4		Group 5		Group 6	
	Adjusted mean	95% CI	Adjusted mean	95% CI	Adjusted mean	95% CI	Adjusted mean	95% CI	Adjusted mean	95% CI	Adjusted mean	95% CI
Pain and stiffness	4.93	4.03–5.84	4.58	3.63–5.53	4.89	3.83–5.95	7.03	6.18–7.88	6.30	4.97–7.63	7.75	6.75–8.74
Activities of daily living	2.29	1.39–3.19	2.61	1.67–3.56	2.62	1.57–3.68	3.93	3.08–4.77	4.69	3.36–6.01	5.10	4.12–6.09
Participation in social activities	1.59	1.13–2.05	1.71	1.23–2.19	1.36	0.82–1.89	2.08	1.65–2.51	2.33	1.65–3.00	1.89	1.39–2.39
General health conditions	1.76	1.51–2.02	1.81	1.54–2.08	1.86	1.56–2.17	2.16	1.92–2.41	2.71	2.33–3.09	2.14	1.85–2.42
Total score	10.6	8.42–12.7	10.7	8.44–13.0	10.7	8.21–13.3	15.2	13.2–17.2	16.0	12.8–19.2	16.9	14.5–19.2

JKOM scores were adjusted for sex, age, duration of symptoms, and knee extension strength

Higher score indicates worse condition

*JKOM* Japanese Knee Osteoarthritis Measure, *95% CI* 95% confidence interval

The comparative analysis of subcategory scores of JKOM for the six subgroups also found that group 6 had the worst score for pain and activities of daily living (ADL) (Fig. 3b, c), indicating that the worst pair of subcategories were more severe radiological change and obesity. On the contrary, group 5 showed the worst score of all groups for social activity and general health (Fig. 3d, e), indicating the weighted contribution of depression to social activity and general health.

We examined the ratios of the use of analgesics and/or oral steroids in each subgroup. The ratios were low (4.8% for analgesics and 1.2% for oral steroids in total) did not yield any significant difference among subgroups (data not shown).

## Discussion

This study identified the factors affecting knee symptoms from several variables using a large community-based cohort including healthy adults. Age, obesity, radiological changes, and depression were consistent factors for knee symptoms. Osteoporosis and inflammation also had some marginal, but undeniable contributions to those. In addition, an exploratory cluster analysis was performed using five factors that affected knee pain and identified six subgroups of knee symptoms in the general population. These six subgroups had different clinical outcomes, with group 6 (moderate ROA with high BMI subgroup) having significantly worse knee symptoms than other groups. This is the first study to use a range of clinical data to comprehensively classify a large number of subjects from the general population, including those with few knee symptoms and mild radiological alterations, into subgroups of knee symptoms and to clarify the weights of the effects on knee symptoms of the relevant clinical domains.

Age and female sex are well-known risk factors for knee pain in patients with knee OA. A unique and controversial result of this study was that the effect of sex on pain was not statistically significant and did not appear to be a distinct feature in cluster analysis. The reason is unknown but may be related to the comprehensiveness of the studies: it may depend more on clinical features such as radiological severity, BMI, depression, and BMD. This point should be investigated further. Although we identified age as a factor influencing knee pain in this study, there is no way to deal with getting older or sex in terms of prevention and treatment. For clinical practice and epidemiologic studies, phenotypic distinctions should be confined to those that affect decisions about treatment or prevention and those that clearly have a fundamental effect on the way we view disease biology and/or disease etiology [9, 18, 19]. Based on this reason, we decided to exclude age from the factors used for cluster analysis.

Although correlation between K/L grade and pain seems to be limited, people with more severe radiographic OA appear more likely to report pain [20]. Our study supported these results by K/L grade showing the highest correlation coefficient with knee pain. In addition, group 4, in which all subjects have K/L grade ≥ 2, showed worth knee symptom than group 1, in which all subjects have K/L grade ≤ 1, indicating that prevention of radiologically defined structural deterioration may, at least partially, alleviate knee symptoms. However, attention should be paid as radiological changes cannot explain the whole symptoms as reported in this study and elsewhere.

BMI significantly affected pain in all models. Also, among the groups showing radiological changes (groups 2 to 6), the high BMI group (group 6) had particularly severe clinical symptoms. The additional sub-analysis gave the same results even after adjusting for possible confounders of age, sex, duration of knee symptoms, and knee extension strength. Interestingly, group 6 reported worse symptoms than group 4, the group with the most severe radiological changes, indicating that obesity has a stronger influence on knee symptoms than the structural alterations represented by radiological changes. A previous study reported that OA pain increases with increasing BMI, even after adjusting for OA severity [21]. Obesity presumably contributes biomechanical stress across the knee joints to affect cartilage degradation. Furthermore, obesity or the presence of excess adipose tissue reportedly induces metabolic inflammation, which would influence knee pain [22, 23], as indicated by the observation that group 6 had higher hs-CRP values than group 4. As recommended by several guidelines, obesity and weight control should be the first factors to be addressed in dealing with knee OA or knee symptoms because they are treatable [1, 24, 25].

Depression, anxiety, or other psychiatric features have been a recent focus because of their influence on pain of the knee or in other musculoskeletal disorders [24, 26, 27]. Indeed, depression significantly affected pain in all models and group 5, the depressive subgroup identified in our cluster analysis, was the subgroup with the second worst symptoms, which is consistent with previous reports and supports the validity of the present study. Surprisingly, the total and pain scores of JKOM showed no significant differences between group 4, the worst radiological change group, and group 5, and scores for social activity and general health were even worse in group 5 than in group 4, indicating that the psychological aspects of the subject may have a similar or greater influence on knee symptoms compared with structural changes. The strong recent emphasis on this aspect suggests that depression or other psychiatric features of each individual should be considered appropriately when dealing with knee symptoms [24].

There have been several reported attempts to classify knee OA or knee symptoms into subgroups or phenotypes. However, few studies have attempted clustering using a multidimensional approach [8]. Kittelson et al. [28] and Knoop et al. [29] attempted to identify phenotypes by data-driven methods using multiple variables. Both studies used the radiographic severity of knee OA, BMI, depression, and lower excess muscle strength as variables for cluster analysis. However, their studies did not include any serological markers or BMD. Another study reported cluster analysis using structural features identified on imaging, including magnetic resonance imaging (MRI) data and clinical symptoms [30]. Although the use of MRI has the advantage of providing detailed structural information, there are concerns on its limited accessibility in clinical practice and even more so in general surveillance. For clinical practice and epidemiologic studies, phenotypic distinctions should be confined to those that affect decisions about treatment or prevention and those that clearly have a fundamental effect on the way we view disease biology and/or disease etiology [9, 18, 19]. We chose variables that are readily accessible in clinical practice, and, therefore, the subgroups identified in this analysis can be applied widely in clinical research and in practice. Also, we chose data-driven approaches, which is recommended in the framework for conducting and reporting OA phenotype research [31].

Several limitations of this study should be acknowledged. First, we analyzed subjects aged over 60 years old in a single ethnic group, the Japanese, and it would be necessary to analyze other groups including younger individuals and/or subjects of other ethnicities to determine whether the clusters identified here can be applied to such populations. Second, when conducting any cluster analysis, the results depend on the variables included. It would be interesting to see whether genetic traits, MRI, or other biomarkers also create distinctive differences. Third, the data for some variables were derived using a single method: depression was assessed by MHI-5 only, and BMD was evaluated solely by calcaneal quantitative ultrasound. Further studies using different methods should be conducted to confirm the results obtained in this study. Finally, the cross-sectional design of our study precludes any conclusions about causal relationships between group characteristics and clinical outcome.

## Conclusions

In summary, this cross-sectional study identified the factors affecting knee symptoms from several variables using a large community-based cohort including healthy adults. In addition, an exploratory cluster analysis was performed using five factors that affected knee pain and identified six subgroups of knee symptoms in the general population. In particular, study participants with worse knee structural damage and with high BMI or depression (groups 5 and 6) reported worse JKOM scores. Because the Nagahama study is a large community-based prospective cohort study, we should be able to accumulate fundamental information useful for developing precision medicine for OA patients by following up the subjects.

## Supplementary Information

**Additional file 1: Supplementary Table 1.** CCC scores of each cluster number. The values of 3 or greater in CCC indicate good clusters. Choosing 6 cluster appeared to be best for this study. CCC; cubic clustering criterion.

## Data Availability

The datasets used and/or analyzed during the current study are available from the corresponding author on reasonable request.

## Abbreviations

ADL: Activities of daily living
BMD: Bone mineral density
BMI: Body mass index
CCC: Cubic clustering criterion
HbA1c: Hemoglobin A1c
HDL-C: High-density lipoprotein-cholesterol
hs-CRP: High-sensitivity C-reactive protein
JKOM: The Japanese Knee Osteoarthritis Measure
K/L: Kellgren/Lawrence
LDL-C: Low-density lipoprotein-cholesterol
MHO-5: Mental Health Inventory-5
NGSP: National Glycohemoglobin Standardization Program
OA: Osteoarthritis
ROA: Radiographic knee OA
SD: Standard deviation
