# Supplementary material for: Obesity with radiological changes or depression was associated with worse knee outcome in general population: a cluster analysis in the Nagahama study
Source: Arthritis Res Ther. 2020 Nov 27;22:280. doi: 10.1186/s13075-020-02375-w (PMC7694310; doi:10.1186/s13075-020-02375-w)
Supplement: Supplementary file 1 — Additional file 1: Supplementary Table 1. CCC scores of each cluster number. The values of 3 or greater in CCC indicate good clusters. Choosing 6 cluster appeared to be best for this study. CCC; cubic clustering criterion. [file 13075_2020_2375_MOESM1_ESM.docx]

Supplementary Table 1. CCC scores of each cluster number

| No. of clusters | CCC | Optimum cluster |  |
| --- | --- | --- | --- |
| 3 | -14.5 |  |  |
| 4 | 9.3 |  |  |
| 5 | 11.5 |  |  |
| 6 | 14.0 | ◎ |  |
| 7 | 7.8 |  |  |

The values of 3 or greater in CCC indicate good clusters.

Choosing 6 cluster appeared to be best for this study.

CCC; cubic clustering criterion
